# Charting the virosphere: computational synergies of AI and bioinformatics in viral discovery and evolution

**DOI:** 10.1128/jvi.01554-25

**Published:** 2025-11-12

**Authors:** Aia Sinno, Ruqaya Baghdadi, Ralph Narch, Serena El Rayes, Sima Tokajian, Charbel Al Khoury

**Affiliations:** 1 Department of Biological Sciences, School of Arts and Sciences, Lebanese American University 37607, Beirut, Lebanon; 2 Department of Biological Sciences, School of Arts and Sciences, Lebanese American University 114792 , Byblos, Lebanon; Universiteit Gent, Merelbeke, Belgium

**Keywords:** virosphere, bioinformatics, artificial intelligence, hybrid workflows

## Abstract

The advancement of metagenomic sequencing has revealed a vast viral diversity while simultaneously exposing limitations of homology-based tools such as BLAST and HMMER, which often fail to detect highly divergent viral genomes. The integration of artificial intelligence (AI) into viromics has transformed this landscape, introducing machine learning and deep learning models—including convolutional neural networks (CNNs), recurrent neural networks (RNNs), and transformers—that extend viral discovery beyond sequence similarity constraints. Structure-based frameworks such as AlphaFold, ESMFold, and Foldseek further enable annotation of divergent viral proteins through conserved 3D folds, while graph neural networks (GNNs) model host-virus interaction and explainable AI enhances interpretability of prediction. Despite their high sensitivity and scalability, AI-driven approaches face notable challenges: computational burden, data set bias, limited explainability, and elevated false discovery rates. This review traces the evolution of computational virology from traditional methods to AI-based and hybrid frameworks. We examine landmark AI tools while underscoring the continuing importance of phylogenetics and functional annotation in contextualizing AI predictions. We propose an integrated workflow that combines AI pattern recognition with classical bioinformatics to enhance both scalability and interpretability. By addressing the limitations of solely AI-driven or traditional approaches, this review presents a unified computational strategy to accelerate viral discovery, enhance evolutionary insights, and strengthen global preparedness for emerging infectious diseases.

## INTRODUCTION

**Fig 1:**
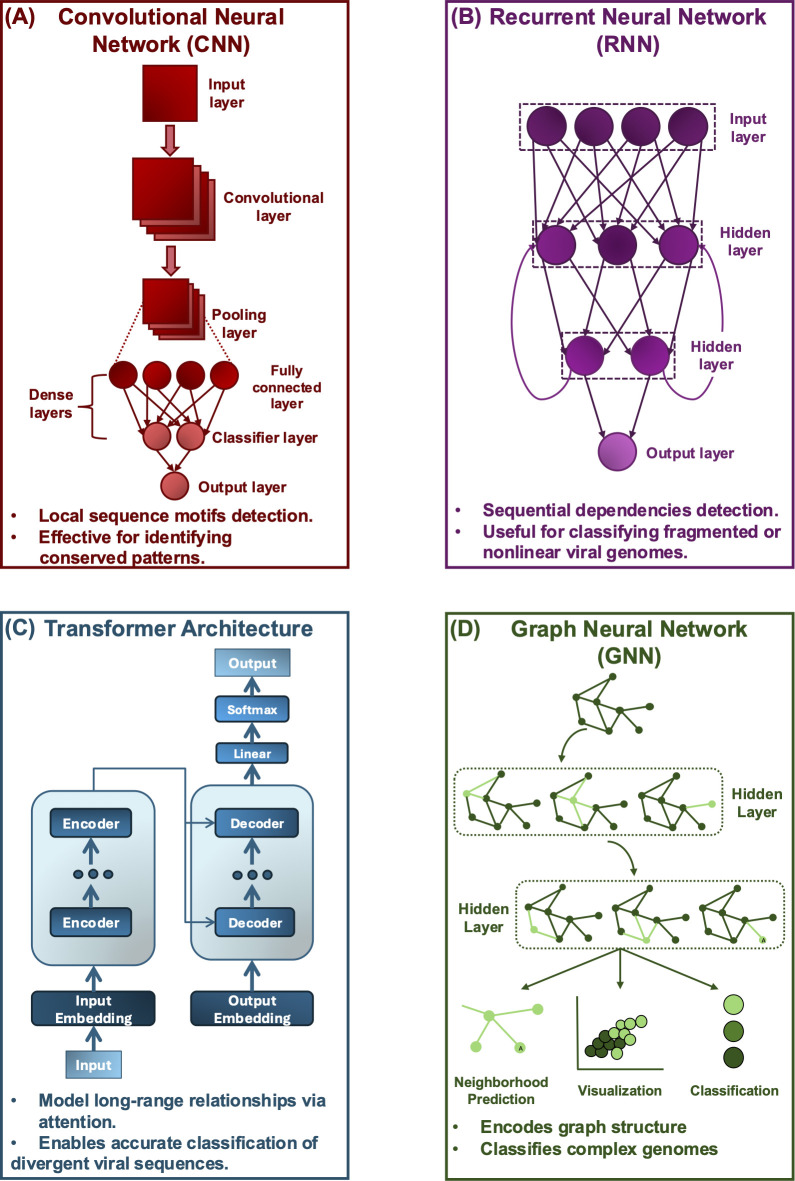
DL architectures underpinning viral genome analysis and host-virus interaction modeling. (**A**) CNNs extract local sequence motifs through convolution and pooling operations, enabling detection of conserved patterns critical for identifying viral genomic signatures. (**B**) RNNs capture sequential dependencies across nucleotide or amino-acid sequences, improving classification of fragmented or nonlinear viral genomes. (**C**) Transformer architectures employ self-attention mechanisms to model long-range relationships across entire genomes, enhancing accurate classification of divergent viral sequences. (**D**) GNNs represent host-virus systems as interconnected graphs, integrating multi-omics and ecological data for neighborhood prediction, visualization, and functional classification of viral interactions.

Deciphering the full extent of viral diversity remains a central challenge in virology (1, 2). Viral genetic plasticity, underpinned by rapid mutation and adaptive versatility, complicates detection, classification, and functional annotation (3, 4). Despite the impact of high-throughput sequencing (HTS) in surveying viral populations, methodological constraints continue to impede both resolution and scope (5). Metagenomics, which directly analyzes genetic material from environmental samples, has expanded the catalog of known viral diversity and ameliorated the detection of novel viral taxa across terrestrial, aquatic, and clinical environments (6). However, fundamental challenges persist: distinguishing viral sequences from host and microbial contaminants, the absence of standardized viral taxonomic frameworks, and substantial computational demands (7). Reliance on sequence homology-based annotation further limits the detection of novel species with minimal reference genome coverage (8). Artificial intelligence (AI) has assumed a transformative role in viromics. Within this framework lies machine learning (ML), a subset of AI comprising algorithms that autonomously improve performance through data-driven training, while deep learning (DL) represents a further specialized form of ML, distinguished by multilayered neural architectures capable of capturing complex processes (9). Convolutional neural networks (CNNs), recurrent neural networks (RNNs), and transformer architecture represent the principal DL frameworks employed in viral genomics (9) (Fig. 1A). CNNs have been originally developed for image recognition that apply convolutional filters to capture local patterns and hierarchical features, rendering them highly effective for detecting sequence motifs in biological data (10). RNNs constitute models in which each computational step is conditioned on prior states, granting the architecture the capacity to preserve temporal or sequential dependencies and thus to compute long-range relationships in nucleotide or amino acid sequences (11) (Fig. 1B). Transformer architectures embody a distinct paradigm, employing multi-head self-attention mechanisms to compute pairwise dependencies across all sequence positions in parallel, thereby affording superior efficiency and scalability in the modeling of global context (12) (Fig. 1C). Together, these architectures constitute the foundation of DL approaches that now drive viral sequence identification, host virus interaction prediction, and the reconstruction of evolutionary trajectories (13). In parallel, structure-based AI methods such as AlphaFold, ESMFold, and Foldseek extend this capability into the structural domain, annotating highly divergent viral proteins through conserved 3D folds (14–16). Unlike traditional similarity-based tools (BLAST and HMMER), these models identify recurring signals in data to infer uncharacterized viral species and support scalable, real-time analysis with minimal reliance on reference genomes (13, 17). Beyond sequence-centric architectures, emerging models such as graph neural networks (GNNs) capture relational information, supporting inference of host-virus interactions and ecological associations through graph-structured data (18) (Fig. 1D).

Building on these computational advances, this review traces the evolution of viral bioinformatics from early metagenomic methods to contemporary AI-driven approaches. We evaluate traditional and AI-based tools, highlighting issues of interpretability, false discovery, and reproducibility. We explore emerging frameworks for explainable AI (XAI) and multi-omics integration, emphasizing the complementary strengths of AI and traditional bioinformatics. Hybrid workflows integrating both approaches will be essential to achieve scalability without sacrificing evolutionary insight or mechanistic understanding, ultimately augmenting the accuracy, scope, and depth of viral diversity analysis beyond current capabilities.

## THE EVOLUTION OF VIRAL DISCOVERY

### Metagenomics: classical paradigms in viral discovery

For much of the 20th century, virology relied on culture-dependent methodologies, necessitating viral replication within host cells. Electron microscopy and serological assays facilitated morphological and antigenic characterization (19), yet these techniques were inherently biased toward cultivable viruses, leaving large portions of the virosphere unexplored. The absence of molecular tools to investigate divergent taxa further limited detection and phylogenetic insight, rendering early viral discovery labor-intensive, low-throughput, and constrained in scope (20, 21). The advent of molecular sequencing in the 1990s, exemplified by BLAST for HTS comparison and phylogenetic inference, marked the beginning of a paradigm shift (22). These advances culminated in the 21st century with a paramount transition: previously unknown viral genomes became accessible directly from clinical samples, circumventing the necessity for viral cultivation. This was illustrated by the identification of human coronavirus HKU1 in nasopharyngeal aspirates through primer-guided Sanger sequencing (23). Early metagenomic efforts employed tools such as Phred, PHRAP, and ClustalW for quality control, assembly, and phylogenetic analysis, revealing unexpected viral diversity within host-associated microbiomes (24–26). A striking example was the recovery of novel viruses from human feces, including exogenous plant viruses within the human gut (27).

The emergence of HTS platforms marked an instrumental pivotal advancement. Early applications of 454 pyrosequencing revealed seven novel RNA viruses in honeybee colonies affected by Colony Collapse Disorder and enabled the characterization of Lujo virus, the first hemorrhagic fever-associated arenavirus identified in Africa (28, 29). Subsequent adoption of Illumina sequencing rapidly eclipsed earlier platforms, owing to its superior base-calling accuracy, depth, and cost-effectiveness (30, 31). In parallel, advances in *de novo* assembly algorithms such as MEGAHIT and SPAdes enabled near-complete reconstruction of viral genomes from highly complex metagenomic data sets, thereby extending virome research beyond fragmentary sequence recovery (32, 33).

Computational pipelines have been equally transformative, converting raw HTS data into meaningful biological insights. Tools such as DIAMOND, BLASTX, and VirSorter2 enhanced sensitivity in homology-based detection, while taxonomic classifiers such as Kraken2 and Kaiju enabled systematic assignment of sequencing reads to known taxa (34–36). To reduce host-derived contamination, alignment-based filtering with Bowtie2 or specialized frameworks such as HoCoRT remained an essential preprocessing step (37, 38). Yet, despite these advances, highly divergent genomes remain refractory to classification, constituting the vast majority of the virome “dark matter” (39, 40). Beyond functional annotation challenges, metagenomic virology demands substantial memory resources and high-throughput processing, prompting the use of cloud-based platforms such as MGnify, IMG/VR, and ViPR for scalable, centralized management of global virome data sets (41–43).

Despite its constitutive role, metagenomics faces inherent constraints. Its dependence on reference-based mapping impedes the detection of highly divergent or uncharacterized viruses, even as it remains indispensable for classifying well-characterized taxa. These computational and methodological confinements have prompted the development of complementary strategies that leverage pattern recognition rather than explicit sequence similarity. In this context, AI has emerged as a transformative approach, capable of detecting highly divergent sequences, inferring functional domains *de novo*, and enabling large-scale automated classification with unprecedented precision. The following sections explore AI’s role in advancing virology beyond the constraints of conventional metagenomic methodologies.

### ML-based approaches

ML approaches have redefined the foundations of viromics by adopting predictive models based on intrinsic sequence features rather than homology-based taxonomies (44). Early applications relied on feature-engineered inputs, most notably k-mer frequency distributions, as probabilistic markers to distinguish viral from non-viral sequences. A fundamental contribution in this space was VirFinder, a logistic regression model trained on k-mer composition features, which outperformed BLAST-based methods in identifying highly divergent viral genomes, including lineages previously inaccessible to homology-dependent tools (45). By circumventing strict reliance on sequence homology, VirFinder facilitated the discovery of novel viral families, although its dependence on predefined feature spaces limited its generalizability to increasingly diverse virome data sets.

Subsequent efforts demonstrated that ML models need not be restricted to k-mer profiles or handcrafted features. Tools such as MarVD2 and GRAViTy exemplify this flexibility, integrating heterogeneous genomic attributes for taxonomic prediction without explicit reliance on k-mers (46, 47). Other ML-driven applications extend to viral binning, prophage prediction, and large-scale taxonomic assignment, highlighting the broader adaptability of ML classifiers across virome analysis (48, 49). Importantly, ML approaches remain indispensable in specific research contexts. Compared to DL, ML classifiers offer greater interpretability and reduced computational burden, attributes that are particularly advantageous in metagenomic virome surveys and hypothesis-driven virological studies where biological transparency is as critical as predictive accuracy (50). In this respect, ML classifiers should not be viewed merely as historical precursors to DL architectures but as enduringly valuable tools that reconcile predictive performance with biological interpretability.

While ML-based approaches have expanded the analytical toolkit of viromics, they remain constrained by the availability of high-quality, labeled trained data sets, which are themselves derived from incomplete and often taxonomically biased viral reference collections (51). Consequently, these models tend to underperform when confronted with novel or ecologically atypical lineages, confining their capacity for broad generalization across environments (52). Feature-engineered inputs, such as k-mer frequencies, further introduce susceptibility to sequencing artifacts and compositional biases, potentially inflating false-positive rates in taxonomic assignments (53). Moreover, the predictive scope of these models frequently diminishes when applied to data sets with divergent nucleotide composition or read-length distributions, necessitating retraining or recalibration. Although ML frameworks are computationally lighter than contemporary DL architectures, their reliance on handcrafted feature spaces and dataset-specific optimization restricts their scalability when deployed across the terabase-scale metagenomic landscapes that increasingly define modern viromics.

## DL FOR SEQUENCE-BASED CLASSIFICATION AND MULTI-OMICS INTEGRATION

DL architectures have fundamentally transformed viral sequence classification by autonomously learning hierarchical genomic features that are inaccessible to traditional bioinformatics and early ML models. Building upon prior ML frameworks, DL models construct hierarchical representations, where early layers detect simple sequence patterns, while later layers autonomously capture complex genomic patterns, thereby enabling the detection of evolutionary novel viral genomes (54).

### CNNs: local feature extraction

CNNs have proven indispensable in capturing proximal sequence motifs within genomic data (55). Through repeated scanning operations (convolutions) across defined sequence windows, CNNs progressively extract local features, facilitating the identification of conserved functional motifs and enhancing taxonomic resolution (10). A prominent example of this approach is DeepVirFinder, which employs multi-layered CNNs to detect fine-grained nucleotide patterns, achieving superior sensitivity in identifying highly divergent viral genomes with minimal sequence homology (13). This capability is particularly critical in metagenomic data sets, where conventional similarity-based tools, namely BLAST and HMMER, often fail to detect novel viral taxa.

Deep-sea metagenomic studies further illustrate the effectiveness of CNN-based approaches. Zhang et al. (56), building on the CNN framework from VirFinder, identified 85,059 viral operational taxonomic units (vOTUs)—the largest catalog of viral taxa reported to date. Eminently, 98.28% of these vOTUs remained unclassified, underscoring the vast extent of viromic “dark matter” and affirming the power of AI-driven approaches to uncover previously unrecognized viral diversity (56). Such findings reveal the effectiveness of CNNs to detect novel viral lineages, particularly within extremophilic environments, such as deep-sea hydrothermal vents, hypersaline lakes, and permafrost, where viral diversity remains largely unexplored.

Despite these strengths, CNNs are inherently limited in modeling long-range dependencies due to their filters focusing only on small regions of a sequence at a time—a property known as the receptive field—and reliance on fixed-size convolutional filters (57). This obstruction is particularly consequential in viral genomes, wherein functional regulation and RNA structures often depend on distal interactions spanning thousands of nucleotides. Coronaviruses exemplify this phenomenon, where long-range RNA–RNA interactions between the 5′ and 3′ untranslated regions play indispensable roles in viral replication and transcription (58, 59). Similarly, in retroviruses such as HIV-1, programmed ribosomal frameshifting is governed by structural and sequence elements separated by extensive nucleotide distances, mandating accurate modeling of distal dependencies (60).

Computational studies have explored strategies to address these limitations. Gupta and Rush (61) demonstrated that a modification called dilated convolutions markedly enlarged the receptive field while circumventing excessive parameter growth, markedly ameliorating the capture of distal genomic dependencies. More broadly, Eraslan et al. (54) emphasized that CNNs excel at proximal motif discovery but struggle to capture higher-order or distant positions in a sequence—so-called long-range dependencies, which instead require hybrid or attention-based architectures. A notable example is the DanQ model, which combines CNNs for local feature extraction with RNNs to integrate sequence-wide context, surpassing convolutional models in regulatory genomics tasks (57).

### RNNs and LSTM: sequential modeling

Building upon CNN architectures and mitigating their limitations, RNNs, particularly their advanced variant, Long Short-Term Memory (LSTM) models, emerged as a potent construct for capturing long-range sequential dependencies within complex viral genomes (62). While CNNs excel at detecting localized sequence features, RNNs process genomic data as continuous streams, enabling the capture of sequence-level variations across entire viral genomes (63). This sequential modeling is particularly important for classifying viruses with noncanonical genome architectures, where nonlinear configurations necessitate computational approaches capable of preserving long-range dependencies across extensive nucleotide spans. An early implementation of this approach, RNN-VirSeeker, leverages LSTM networks to model these long-range relationships, overcoming the structural and contextual fragmentation that often limits CNN performance when classifying highly mutational and discontinuous viral genomes (64). LSTM architectures progressively curtail the vanishing gradient problem—a training limitation where simpler models gradually lose long-term information—thereby allowing the retention and propagation of critical evolutionary and structural information throughout the learning process.

The hierarchical design of this model has led to significant advancements in viral bioinformatics, particularly in three key areas: (i) retaining essential sequence information over long genomic distances, enabling the identification of highly mutagenic viral taxa; (ii) characterizing genome-wide structural variations, which is critical for understanding viral recombination, antigenic drift, and host-specific adaptation; and (iii) improving recall accuracy for truncated viral contigs (<500 bp), where conventional CNN-based models often struggle due to the fragmented nature of metagenomic reads. Benchmarking studies have consistently demonstrated the superior performance of RNN-VirSeeker compared to its predecessor, VirFinder, establishing it as a leading AI-based model for the classification of truncated viral genomes (64). These findings highlight the importance of modeling temporal dependencies in viromic taxonomic classification, reinforcing the critical role of DL architectures in resolving the complexity of viral genome evolution and phylogenetic diversity.

The ongoing advancement of DL is expected to give rise to hybrid architectures in which CNNs and RNNs are complemented by attention-based or transformer modules, enabling the extraction of local motifs, sequence-wide dependencies, and global contextual relationships within viral genomes (65). Rather than supplanting CNNs or RNNs, these transformer-enhanced architectures build upon the strengths of existing models, integrating specialized modules for motif detection, sequential modeling, and long-range contextual inference (66). As metagenomic research continues to expand our understanding of the global virosphere, AI-driven tools are set to play a central role in pandemic forecasting, antiviral drug discovery, and tracking viral transmission, making them key elements of modern bioinformatics and computational virology.

### Transformer-based AI models: genome-wide contextual encoding

Transformer-based architectures represent a pivotal advancement in computational virology, overcoming the inherent limitations of CNNs and RNNs while complementing their specialized capabilities by enabling comprehensive, genome-wide contextual encoding with unparalleled efficiency and accuracy (67). Leveraging multi-head self-attention mechanisms, transformers capture long-range dependencies across viral genomes, permitting tasks such as viral taxonomy, host-virus interaction prediction, and the functional annotation of novel viral taxa (12). These competencies are particularly valuable in metagenomic virome analysis, where traditional classifiers often struggle to resolve fragmented genomes, disambiguate highly divergent lineages, or distinguish viral sequences from microbial contaminants. Unlike RNNs, which analyze sequences step by step, transformers employ parallel computation across all sequence positions at once, rendering them faster for large data sets (68). However, this advantage does not eliminate their considerable computational burden, which remains a critical bottleneck for applications such as AlphaFold2 and large-scale viromics studies (69, 70).

At the forefront of transformer-based advances is LucaProt (2023), trained on an extensive 51-terabase metagenomic sequencing data sets and identified 161,979 novel virus species (12). Its innovation lies not in incremental performance metrics but in methodological shifts: utilizing self-supervised learning, where the model discovers patterns directly from raw data without the need for manual labeling; integrating proteomic sequences with structural topology; and eliminating reliance on manually aligned data sets (12). These features allow LucaProt to infer evolutionary lineages, detect cryptic homologous sequences, and annotate functional domains such as RNA-dependent RNA polymerase (RdRp) domains (12).

**Fig 2:**
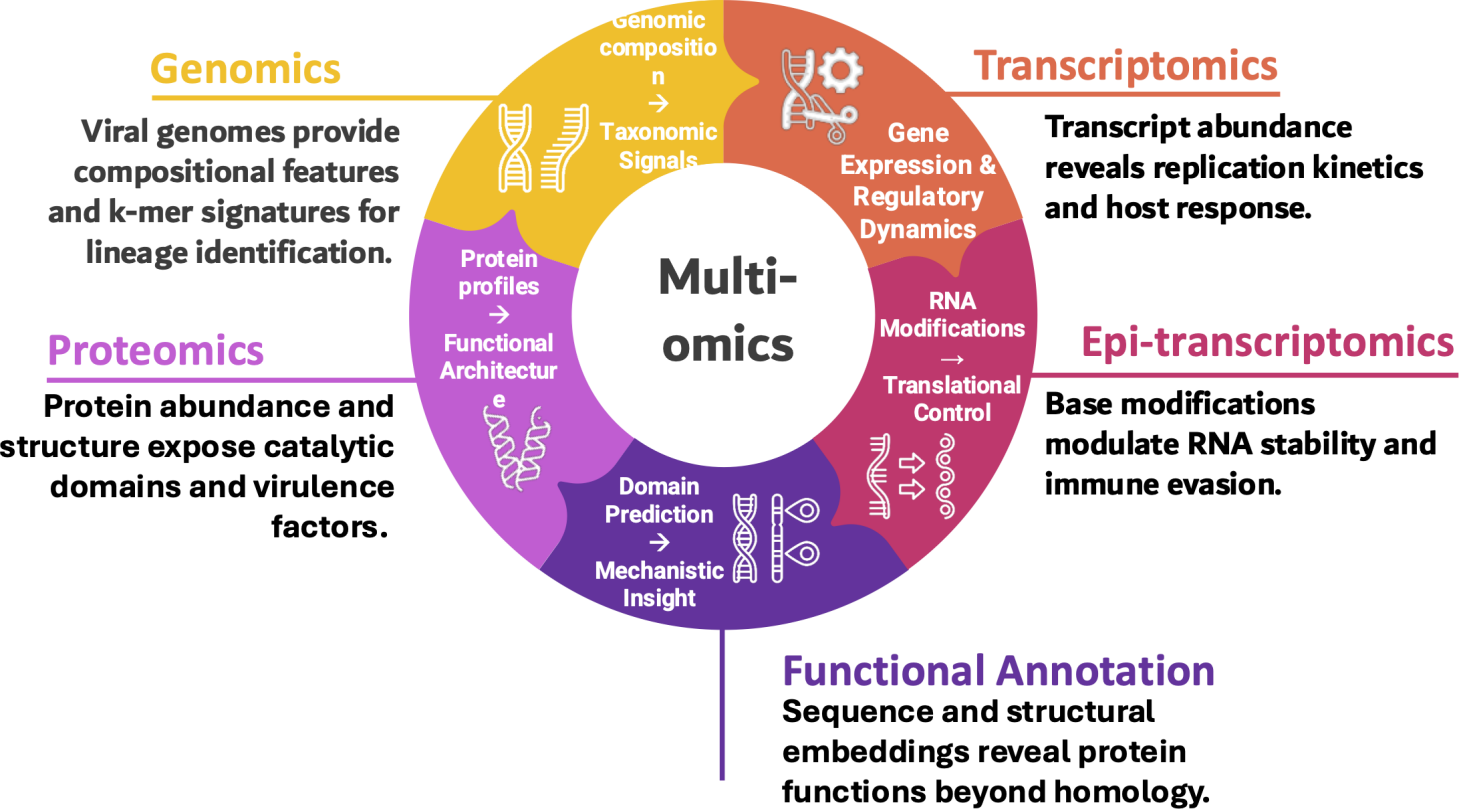
Multi-omics integration enhances functional and evolutionary insight in viral systems. Conceptual overview of multi-omics approaches used to contextualize AI-driven viral discovery. Genomics provides compositional and k-mer signatures for lineage identification and taxonomic placement. Transcriptomics captures replication kinetics and host-response patterns through differential gene expression. Epi-transcriptomics adds a regulatory layer, where RNA base modifications influence transcript stability and immune evasion. Proteomics resolves catalytic domains and virulence factors through protein abundance and structural profiling. Functional annotation, aided by sequence- and structure-based embeddings, predicts domain architectures and mechanistic roles beyond homology. Together, these complementary layers enable integrative modeling of viral function, evolution, and host interaction within a unified computational framework.

Beyond taxonomic classification, transformers have become central to hybrid AI-virology pipelines, supporting real-time surveillance, high-resolution epidemiological forecasting, and mechanistic reconstruction of host-virus interactions (71). The integration with multi-omics technologies (metatranscriptomics, proteomics, and epitranscriptomics) enables functional characterization of viral genes, identification of novel RNA modifications, and prediction of host-viral interaction, thereby revealing the molecular underpinnings of viral evolution and cross-species transmission (Fig. 2). Transformers complement existing CNN and RNN modules, allowing hybrid pipelines to combine local motif detection, sequential modeling, and genome-wide attention for maximal analytical resolution.

Notwithstanding their transformative potential, transformer-based architectures are not without critical challenges. Their effectiveness is predicated upon access to massive training corpora—often measured in terabases of sequence data—which not only sustains prohibitive computational and financial costs but also risks embedding dataset-specific biases that skew downstream inference (72). These models, while offering unparalleled parallelization and scalability in principle, remain computationally intensive in practice, demanding high-performance GPU or TPU infrastructures that are inaccessible to many laboratories (72). Furthermore, the interpretability of transformer outputs remains limited; their self-attention mechanisms, though powerful, often operate as opaque black boxes (BBs), complicating efforts to trace predictive outcomes to biologically meaningful features (73). These constraints underscore a paradox intrinsic to transformers: while they scale efficiently to large data sets, their development and deployment are resource-intensive, and their predictions frequently necessitate complementary validation through phylogenetic or structural benchmarks to ensure biological plausibility.

While the sequence-based methods continue to dominate virological discovery, they capture only parts of AI’s transformative impact; in parallel, a structural revolution, led by AlphaFold and its successors, has redefined protein fold prediction and extended viral annotation into the 3D domain.

### Structure-based AI in viromics: the rise of AlphaFold and successors

Structure-based AI has revolutionized protein conformation prediction, offering an orthogonal lens for functional inference in protein biology. Three key tools exemplify this shift: AlphaFold, which pioneered near-experimental accuracy in protein structure prediction from sequence alone (14); ESMFold, which scales this capability across millions of sequences using transformer-based language models (15); and Foldseek, which enables ultra-fast structural comparisons at the proteome scale (16). In viromics, these advances are particularly impactful as divergent viruses often evade detection due to minimal sequence similarity with known taxa. Yet, their encoded proteins frequently retain conserved structural scaffolds, such as the canonical folds of RdRp, helicases, and capsid proteins, that remain detectable through AI-driven structure prediction (74). This structural anchoring enables functional annotation and taxonomic placement of proteins that would otherwise remain uncharacterized. Already, such approaches have revealed previously unrecognized viral clades and expanded the known evolutionary diversity of RNA viruses.

Beyond taxonomy, structure-based AI unlocks mechanistic insights inaccessible to sequence-based methods. Predicted receptor-binding domains can offer early indicators of zoonotic potential, while structural reconstructions of polymerase active sites inform antiviral drug design (75, 76). Crucially, ESMFold’s capacity to scale across terabase-scale metagenomic data sets, combined with Foldseek’s rapid search capabilities, now makes it feasible to embed structure-based annotation into real-time viral surveillance pipelines—transforming how we interpret, classify, and respond to the virosphere. As these increasingly powerful models redefine viral discovery, an equally pressing challenge arises: ensuring that their predictions are interpretable, reproducible, and ethically accountable, a task addressed by XAI.

### XAI and ethical imperative in viromics

As structure-based AI tools become increasingly central to virological research, so too must our capacity to interrogate the rationale behind their predictions. This imperative has catalyzed the emergence of XAI, a set of frameworks designed to render DL models transparent, interpretable, and biologically trustworthy (77). While complex neural architectures have demonstrated remarkable predictive power, they often produce outputs devoid of explicit reasoning. This lack of interpretability can erode confidence in their biological validity—particularly in viromics, where such models are applied to taxonomic classification, virus discovery, and cross-species transmission analysis. In such contexts, opaque predictions risk misinterpretation and hinder downstream utility.

XAI addresses this challenge across multiple strata of model architecture. At the input level, attribution methods such as integrated gradients, DeepLIFT, and SHAP quantify the contribution of individual nucleotides or amino acids to a given prediction (78–80). Within intermediate layers, attention visualization and saliency mapping reveal which genomic or structural regions are emphasized during inference (81). At the decision layer, surrogate models and rule-extraction techniques distill complex outputs into simplified human-readable logic approximations (82). These interpretability tools not only clarify model behavior but also support error detection, hypothesis generation, and generalizability. For instance, feature attribution may expose overreliance on sequencing artifacts or dataset-specific biases; attention heatmaps can highlight previously uncharacterized genomic regions warranting functional investigation; and transparency in model focus helps ensure that insights generalize across data sets rather than reflect overfitting.

The ethical dimension of interpretability is equally critical. When AI-derived predictions inform public health policy or real-time surveillance, stakeholders must be able to audit and reproduce results with confidence in their biological plausibility. Emerging XAI paradigms further extend interpretive capacity. Prototype-based networks and concept bottleneck models explicitly link internal representations to known viral functions (44), while counterfactual explanations reveal how perturbations to sequence or structure alter model outputs (83). Multimodal XAI approaches, which integrate sequence, structure, and host-interaction data, offer a more holistic interpretive framework suited to the complexity of viral systems (84). For XAI to fulfill its promise, however, the field must establish community standards and benchmark data sets for interpretability, enabling rigorous cross-comparison and fostering trust among laboratories and applications.

With these explainability frameworks in place, AI models evolve from opaque predictors to interpretable systems—laying the groundwork for more advanced relational modeling. GNNs, which capture host-virus interactions and ecological relationships, represent a natural extension of this trajectory. Their integration of multi-omics and structural data benefits equally from the transparency afforded by XAI, enabling biologically grounded inference at network scale.

### GNNs: multi-omics and relational modeling

To overcome the limitations of feature-engineered models, GNNs, which treat viral and host data as networks of nodes and connections rather than isolated sequences, have emerged to capture the relational architecture of viral ecosystems. GNNs, with PhaGCN as a prominent exemplar, enable systems-level interpretation of the virome by leveraging graph-theoretic embeddings—vector representations that capture viral and host features to model their relationships beyond the analytical scope of sequence-centric classifiers (85). Departing from conventional classifiers that evaluate viral sequences in isolation, GNNs embed viral genomes, host-derived sequences, ecological metadata, and taxonomic relationships within network representations. This design permits inference of latent functional associations and ecological interactions within host-virus relationships, thereby surmounting many limitations of linear sequence analysis (85). Such methodological advances have proven especially valuable for zoonotic surveillance, where predicting cross-species transmission demands models that accommodate recombination, episodic host shifts, and the ecological context that obscures simple phylogenetic signals (86). Tools like PhaGCN demonstrate how genomic and ecological features can be integrated to improve host-viral interaction prediction relative to models that rely solely on sequence homology or phylogenetic proximity (85).

Beyond host prediction, GNNs have become instrumental for classifying viral ecological networks within complex metagenomic data sets drawn from marine, soil, and vector-borne communities (67). Applications of PhaGCN in global virome studies have revealed previously undetected host-virus associations across diverse ecosystems, underscoring the ability of graph-structured models to illuminate transmission dynamics and ecological modularity that elude linear sequence methods (85).

Although fully integrated virus-centric GNN pipelines that jointly assimilate viral/host sequence embeddings with multi-omics layers are still nascent, there is a clear precedent in adjacent biomedical domains. Modality-aware GNN frameworks (MOTGNN) have successfully combined mRNA, miRNA, and DNA-methylation layers via interpretable graph embeddings to augment classification and biomarker discovery, demonstrating the technical feasibility of multi-omics + GNN integration (87). These methodological precedents provide a ready blueprint for analogous viromics efforts, where transcriptomics, proteomics, and epitranscriptomics could be fused with sequence-derived embeddings to enhance host-specificity prediction, zoonotic risk scoring, and functional annotation.

Looking forward, GNNs, especially when complemented with transformer-derived sequences and other deep representations, promise to sharpen host-specificity prediction, reveal ecological drivers of transmission, and resolve phylogenetic connectivity across the expanding virosphere. As metagenomic and multi-omics data sets continue to grow, graph-based DL will become an increasingly indispensable component of systems-level viromics, complementing sequence-based DL models and enabling richer inference of viral evolution and epidemiological emergence.

## BRIDGING AI AND TRADITIONAL BIOINFORMATICS IN VIROMICS: HYBRID WORKFLOWS

AI-driven methods and traditional bioinformatics constitute a scalable and grounded framework as complementary components of integrated workflows. AI excels at detecting highly divergent viral sequences in large-scale metagenomic data sets, while traditional bioinformatics provides the evolutionary context needed to validate and interpret these validations (Table 1). A representative example is LucaProt, which has demonstrated the capacity to recover highly divergent RNA viruses from complex environmental metagenomes, revealing lineages inaccessible to conventional workflows (12, 88). However, without rigorous phylogenetic reconstruction, the taxonomic classification of such sequences remains conjectural. Multiple sequence alignment programs (e.g., MAFFT and Clustal Omega) serve as indispensable preprocessing tools that generate homologous positional matrices for comparative analysis, yet they do not themselves constitute phylogenetic inference (89, 90). Evolutionary reconstruction must instead be resolved with statistically robust tree-building algorithms, with IQ-TREE emerging as a de facto standard owing to its integrated model selection, likelihood optimization, and branch-support testing (91). Depending on data set scale and analytical practices, alternatives such as RaxML-N, PhyML, or FastTree 2 may be employed (92–94). Tool selection is also shaped by domain-specific contexts. For example, dedicated frameworks such as VIRIDIC—commonly employed in bacteriophage and archaeal virus taxonomy—provide complementary methodologies aligned with established community standards (95), while network-oriented frameworks such as vConTACT2 and VICTOR are widely used to interrogate complex viral assemblages (96, 97).

**TABLE 1:** A comparative evaluation of methodologies in virology

Feature	Traditional bioinformatics	AI-based approaches
Virus identification	Based on sequence similarity searches (e.g., BLAST, HMMER, tBLASTx), requiring close homologs in existing databases	Detects viruses using learned sequence patterns, allowing classification of highly divergent viral genomes even in the absence of sequence homology (CNNs, transformers)
Ability to identify novel viruses	Limited by reference databases; fails to detect highly mutated or previously unknown viral species	Can infer viral genomic features *de novo*, recognizing unknown viruses with no prior homology, dramatically increasing discovery rates in uncharacterized viromes
Computational efficiency	High processing time for large-scale metagenomic data sets due to sequential similarity searches	Optimized processing with parallel computation, significantly reducing the time required for genome annotation and classification (e.g., LucaProt outperforms BLAST in large-scale virome discovery)
Scalability	Struggles with massive sequencing data sets due to reliance on homology searches and alignment tools, leading to computational bottlenecks	Highly scalable, leveraging DL architectures (CNNs, RNNs, transformers) that can process terabase-scale virome data sets in real time
Interpretability	Rule-based classification makes results easier to validate, providing clear phylogenetic relationships and functional annotations	"Black-box" problem: AI models lack interpretability, making it difficult to trace decision-making processes (e.g., why a model classified a sequence as viral); XAI frameworks are under development
False positives/negatives	Lower false discovery rates due to stringent homology-based filters, but limited sensitivity, leading to underestimation of viral diversity	Higher sensitivity in detecting novel viruses but prone to false positives, necessitating validation through independent virological assays
Functional annotation	Requires homology-based domain predictions using tools like InterProScan, Pfam, and HMMER, limiting insights for novel viruses with uncharacterized functional domains	AI models can infer functional domains without prior reference, using protein structure prediction and deep-learning classifiers, providing new insights into viral gene functions
Virus–host interaction prediction	Based on phylogenetic host–virus relationships or manually curated interaction databases	GNNs like PhaGCN model virus–host interactions using network-based learning, outperforming traditional host-prediction models
Metagenomic complexity handling	Requires manual filtering of host and bacterial sequences before viral annotation	AI models automatically classify viral contigs, reducing the need for extensive manual curation (e.g., VirSorter2, VirHunter)
Multi-omics integration	Limited compatibility with transcriptomic, proteomic, and epigenomic data	AI models can integrate multi-omics data, including metatranscriptomics, proteomics, and structural genomics, to improve viral discovery
Adaptability to novel data	Slow to update, as it relies on curated databases that must be manually revised	AI continuously learns from new sequencing data sets, allowing for rapid adaptation to newly emerging viral taxa
Structure-based discovery	Limited to experimentally solved structures, often unavailable for viral proteins	Structure-prediction tools (AlphaFold, ESMFold, Foldseek) enable annotation of divergent proteins through predicted 3D conformations

**Fig 3:**
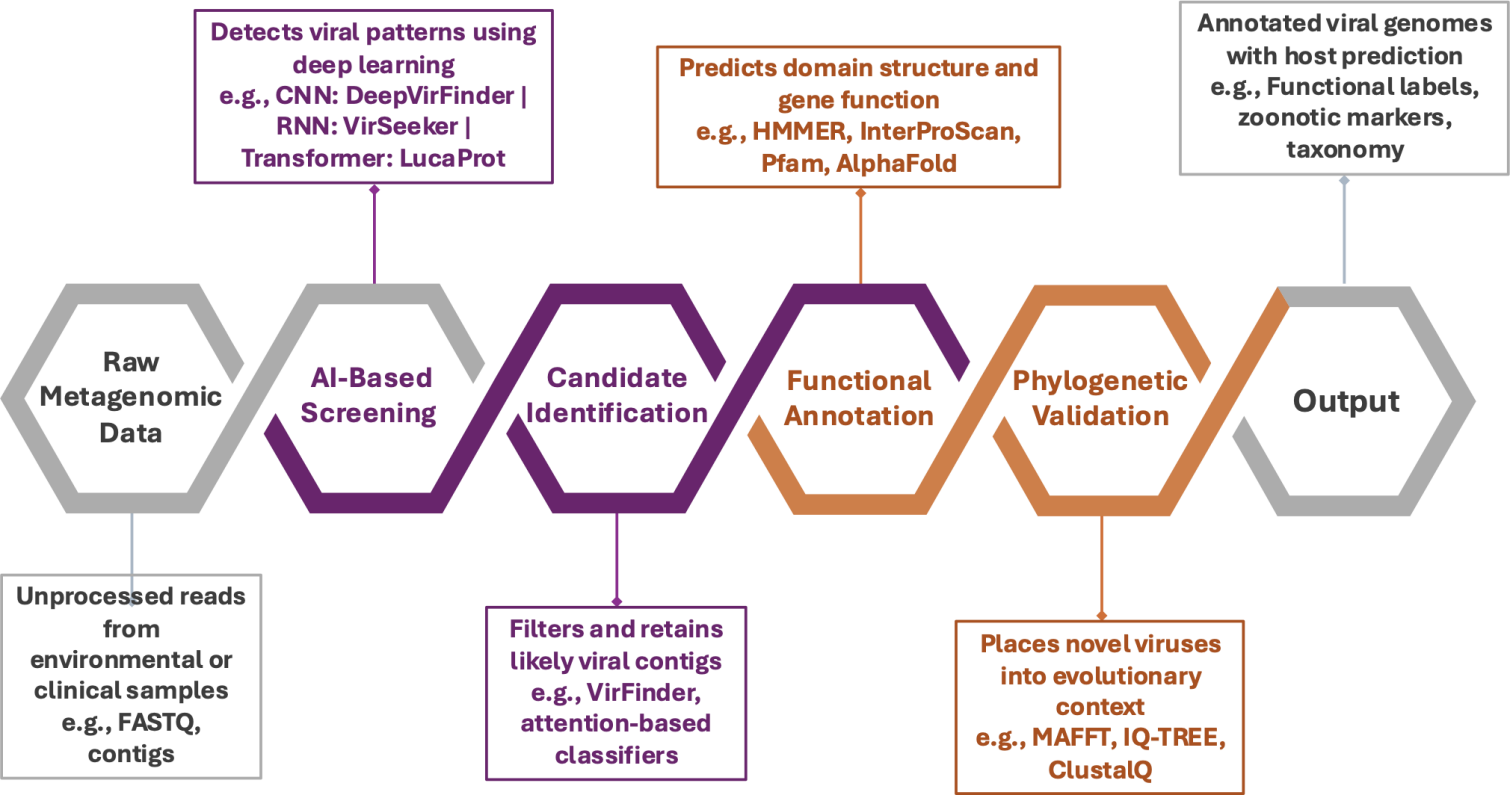
Hybrid AI–bioinformatics workflow for viral genome discovery and annotation. Schematic representation of an integrated computational pipeline combining AI-based screening with classical bioinformatics for viral genome annotation. Raw metagenomic reads from environmental or clinical samples are first processed through AI-based screening (e.g., CNN: DeepVirFinder, RNN: VirSeeker, Transformer: LucaProt) to detect viral sequence patterns. Candidate identification filters and retains likely viral contigs using probabilistic classifiers (e.g., VirFinder). Functional annotation follows, predicting gene function and domain structure with established bioinformatic tools (e.g., HMMER, InterProScan, Pfam, AlphaFold). Phylogenetic validation places novel sequences within an evolutionary framework using multiple sequence alignment and tree-building algorithms (e.g., MAFFT, IQ-TREE, ClustalW). The pipeline yields annotated viral genomes with functional labels, host associations, and zoonotic markers, thereby enhancing biological interpretability while preserving AI-driven scalability.

By integrating AI-based predictions with classical tree-building methods, researchers can trace the evolutionary origins of newly identified viruses, thereby strengthening the accuracy of their taxonomic placement. In this complementary framework, AI functions as a discovery tool, while traditional bioinformatics serves as a taxonomic arbiter indispensable for refinement through phylogenetic inference and evolutionary contextualization (Fig. 3).

## AI AS A CATALYST FOR BIOINFORMATICS EFFICIENCY

**Fig 4:**
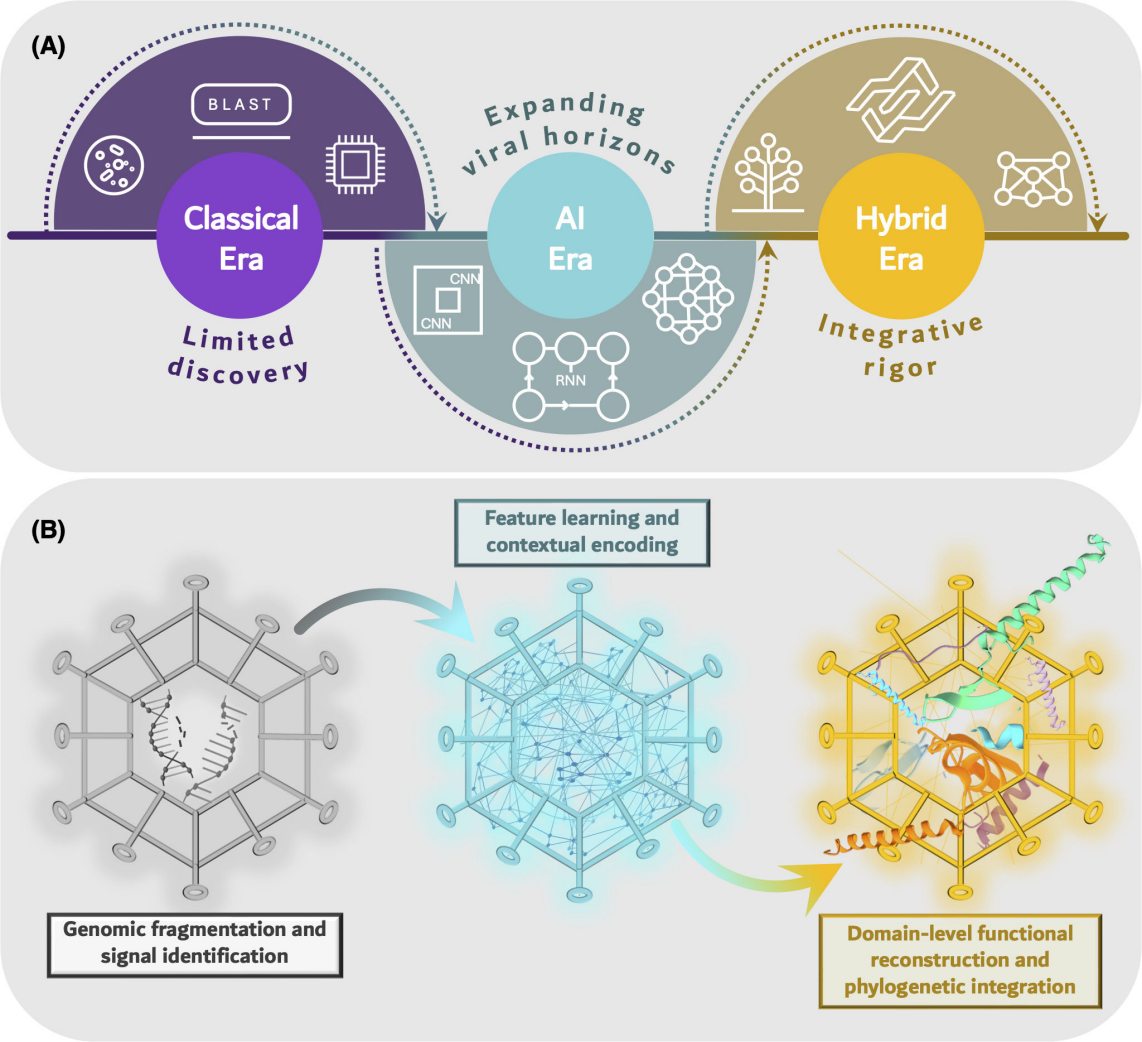
Hybridization of AI and classical bioinformatics paradigms in viromics. (**A**) Depiction of the methodological evolution from traditional discovery frameworks limited by exhaustive similarity searches toward AI-augmented and hybrid approaches that enable scalable and targeted viral identification. (**B**) Representation of the hybrid computational pipeline wherein AI-driven sequence prioritization precedes domain annotation and phylogenetic inference, exemplifying a synergistic model that optimizes bioinformatics efficiency while maintaining analytical rigor.

A major limitation in conventional viromics lies in the substantial computational burden imposed by similarity-based search algorithms, a challenge amplified by the vast scale of modern metagenomic data sets. AI offers the capability to prioritize sequences for downstream bioinformatics analysis, thereby significantly reducing the time required for annotation workflows (Fig. 4A). Instead of applying BLAST indiscriminately across millions of reads, AI frameworks enable the preliminary curation of data, guiding traditional tools toward sequences of highest virological relevance. As an initial filter, a CNN-based classifier can remove bacterial and host-derived contaminants from raw sequencing reads, as it demonstrates promise in taxonomic classification of metagenomic reads (98). The refined data set is then subjected to domain annotation using HMM-based approaches and phylogenetic clustering, enabling the delineation of conserved domains and the inference of evolutionary relationships (99). This approach, both rigorous and targeted, ensures the strategic application of bioinformatics tools, enhancing computational efficiency while preserving analytical accuracy (Fig. 4B).

## IMPROVING AI INTERPRETABILITY USING BIOINFORMATICS BENCHMARKS

A key limitation of AI models lies in their interpretive opacity, the BB phenomenon, wherein the rationale behind predictive outputs remains inaccessible to human understanding. Training AI models on biologically curated data sets helps reduce this ambiguity and enhances explanatory robustness. This hybrid framework, combining the predictive capacity of AI with the interpretability of evolutionary analysis, addresses the knowledge-based disconnect in BB models. For instance, transformer-based models such as LucaProt infer viral genes through contextual pattern recognition, enabling annotation within genomically unexplored viral sequences (12). Yet in many cases, AI-predicted sequences remain functionally ambiguous, lacking empirical or mechanistic validation. In such instances, a triad of bioinformatic tools (Pfam, InterProScan, and structural modeling via AlphaFold) becomes indispensable for determining whether these putative viral genes encode domains consistent with known viral functions (100–102). The reciprocal interplay between AI-driven discovery and bioinformatics-based validation systematically reduces misclassification, as the integration of biologically validated annotations progressively enhances the reliability of interpretation of AI models.

## STRENGTHENING FUNCTIONAL ANNOTATION THROUGH COMBINED APPROACHES

AI-driven models can predict functional domains in viral proteins by leveraging statistical patterns rather than evolutionary precedent. Yet such predictions, while computationally useful, often suffer from ambiguous or non-specific classifications. In contrast, traditional bioinformatic methodologies, anchored in rigorously curated domain taxonomies, offer clarity about the process but often falter when challenged with entirely novel viral proteins. By combining AI-based reasoning with function-based annotation, a hybrid approach supports more accurate and reliable predictions of protein function across different levels of biological understanding. For example, an AI-identified viral protein can be further examined using HMMs, where conserved motifs may reveal similarity to known functional domains (103). Should homology searches fail to uncover related sequences, protein structure modeling offers an alternative approach to understanding by aligning predicted folding architectures with those of known viral enzymes. Integrating AI-driven predictions with structural validation provides a strong foundation for assigning functions to previously unannotated viral proteins, such as polymerases, capsid components, and host-pathogen interaction factors.

## OPTIMIZING VIRUS-HOST INTERACTION PREDICTIONS WITH AI AND PHYLOGENETICS

Traditional approaches to predicting host-virus interactions have relied heavily on phylogenetic proximity or manually curated host-association data sets. While structurally sound, these methods are limited by their dependence on existing taxonomies and narrow genomic coverage. Recent advances, particularly through GNNs such as PhaGCN, have introduced network-based learning frameworks that extrapolate host range and transmission dynamics from hidden patterns in viral sequences (85). Nevertheless, these algorithmic predictions require molecular and evolutionary validation through experimental data. Combining AI-generated insights with independent phylogenetic analysis creates a stronger framework for understanding host-virus adaptation. For example, an AI model might flag a novel viral sequence as having zoonotic potential. Validating this by comparing its receptor-binding regions to known host receptors adds structural evidence, linking AI predictions to real biological function (104).

## AI-DRIVEN MULTI-OMICS INTEGRATION: AIDING BIOINFORMATICS IN COMPLEXITY REDUCTION

Earlier viromic approaches relied mainly on genomic data, but AI-enabled systems have introduced a more integrated model, enabling combined analysis of metagenomic, transcriptomic, and proteomic data. Metatranscriptomics reveals viral gene activity within microbiomes, though separating viral from host RNA remains a major analytical challenge (105). By using AI algorithms to pre-classify viral reads, differential expression analysis becomes more targeted and effective in uncovering host-pathogen interactions. When workflows are designed to be iterative and connected, AI and bioinformatics work together as a unified system, making complex problems more manageable and turning large virome data sets into biologically meaningful insights (7) (Fig. 3).

## CONCLUSION

Progress in viromics relies not on choosing between AI and traditional bioinformatics but on combining their strengths within a shared framework. AI brings speed, sensitivity, and scalability, making it well-suited for identifying viral sequences in large, complex data sets. Yet, its real value unfolds when these predictions are validated and refined using established bioinformatics tools that provide evolutionary, structural, and functional context. This integration is essential: while AI can detect patterns that suggest novel viruses or host associations, bioinformatics methods are needed to interpret those signals and confirm their biological relevance. This combined approach enables a more complete and meaningful analysis of viral diversity and function.

Looking forward, several priorities ought to shape the field. First, hybrid workflows that incorporate both AI and bioinformatics will allow researchers to adapt as new tools and data emerge. The development of modular pipelines and community-endorsed benchmarks will ensure interoperability and reproducibility across laboratories. Second, interpretability frameworks—from feature attribution and saliency mapping to concept bottleneck models and counterfactual reasoning—will affirm that predictions remain transparent and biologically grounded. By clarifying which sequence motifs or structural domains drive model outputs, XAI strengthens biological plausibility and facilitates experimental validation. Third, the integration of structure-based AI methods will continue to expand viral annotation beyond sequence homology, enabling classification of highly divergent proteins through conserved 3D folds and illuminating mechanistic features critical for understanding viral function. Shared data sets and reproducible pipelines will promote collaboration and transparency, accelerate discovery and improve our ability to monitor and respond to viral threats.

Investment in comprehensive training programs will collectively certify accessibility, empowering researchers across diverse settings to leverage stated computational advances. Promoting collaborative consortia and advancing transparent data sharing will accelerate the translation of computational innovations into practical applications, including real-time viral surveillance, rapid pathogen identification, and the design of targeted antiviral therapeutics. Ultimately, these integrated and accountable approaches will not only advance fundamental virological research but also strengthen global capacity to monitor and respond to emerging viral threats. In this manner, the convergence of AI and traditional bioinformatics promises to enrich our comprehension of viral evolution, host specificity, and the overarching architecture of the global virosphere, positioning computational viromics as an indispensable tool for both basic research and public health preparedness.
